# Evaluation of microsurgical versus conventional periodontal flap procedures: A prospective randomized comparison

**DOI:** 10.6026/973206300223352

**Published:** 2026-06-30

**Authors:** Amit Kumar Upadhyay, Megha Modi, Chandresh Kumar Shukla, Priyanka Jha, Prachi Kapade, Swarnabha Halder

**Affiliations:** 1 Department of Periodontology, Shri Balaji Institute of Dental Sciences, Raipur, Chhattisgarh, India; 2 Department of Oral and Maxillofacial Surgery, New York University College of Dentistry, New York, United States; 3 Department of Orthodontics, People's College of Dental Sciences and Research Centre, Bhopal, Madhya Pradesh, India; 4 Department of Periodontology, New Horizon Dental College and Research Institute, Bilaspur, Chhattisgarh, India; 5 Department of Oral Pathology and Microbiology, MGV KBH Dental College and Hospital, Nashik, Maharashtra, India

**Keywords:** Periodontal flap surgery, microsurgery, conventional surgery, clinical attachment level (CAL), probing pocket depth (PPD), randomized controlled trial (RCT), periodontitis

## Abstract

Comparative effectiveness of microsurgical versus conventional flap surgery in periodontal treatment remains inadequately defined. Therefore, it is of interest to compare clinical outcomes of microsurgical and conventional periodontal flap procedures in 100 patients with chronic periodontitis. Hence, patients were allocated to two groups, with assessment of probing pocket depth (PPD), clinical attachment level (CAL), gingival index (GI), plaque index (PI), post-operative pain and healing at baseline, 1 month and 3 months. The microsurgical group showed greater reduction in PPD, higher CAL gain, improved healing and reduced post-operative discomfort compared to the conventional group (p<0.05). Thus, we show superior clinical and patient-centered outcomes with microsurgical techniques, supporting their use in periodontal therapy.

## Background:

Periodontal diseases are long term inflammatory diseases that affect the supporting of teeth and are a significant cause of tooth loss in the world. Microbial plaque is the main etiological agent and it triggers a host-mediated inflammatory reaction and results in the destruction of periodontal tissues, such as the gingiva, periodontal ligament and alveolar bone [1]. Traditional periodontal treatment is aimed at removing etiologic factors and reducing periodontal pockets by the use of both non-surgical and surgical procedures. Periodontal flap surgery is among the surgical procedure that is important in offering access to debridement and periodontal regeneration [2]. The traditional periodontal flap protocol entails reflection of the gingival tissues so that the direct visualization and instrumentation of the root surfaces and osseous defects can be effected. Although this method is effective, it is also commonly linked to some shortcomings, such as the increased tissue trauma, post-operative pain and slow healing [3]. Such shortcomings have led to the search of minimally invasive procedures to enhance surgical accuracy and patient outcomes. Periodontal microsurgery has been an emerging high-tech form of surgery, involving the use of magnification equipment, like surgical microscopes or loupes, specifically designed microsurgical tools and smaller sutures in recent years [4]. The idea of microsurgery in periodontics was brought about to increase the visual acuity, precision and decrease tissue trauma. Improved visibility and magnification facilitate clinicians to do delicate surgeries more precisely and achieve better wound approximation and better healing outcomes [5]. The principles of gentle tissue treatment, passive wound closure and the atraumatic suture techniques underlie microsurgical techniques. These guidelines help to decrease the inflammatory process, accelerate recovery and enhance esthetic outcomes [6]. A number of studies have shown that microsurgical techniques may result in better clinical results especially in surgeries like root coverage, regenerative therapy and implant surgery [7]. Although periodontal microsurgery has been on the rise of interest, little is known about its use in the context of standard flap surgery. The literature available on most part is about specific procedures and not general periodontal flap surgery. Furthermore, high-quality evidence directly comparing microsurgical and conventional modalities on the basis of clinical parameters (reduction in probing depth, gain in attachment and patient-centered outcomes) is limited [8]. The need for evidence-based evaluation of these techniques is essential to guide clinical decision-making. Randomized controlled trials are regarded as the gold standard of evaluating the effectiveness of therapeutic interventions because they reduce bias and offer credible findings [9]. Therefore, it is of interest to establish whether the microsurgical technique has important benefits over the traditional technique in periodontal therapy.

## Materials and Methodology:

## Study design:

This study was designed as a randomized controlled clinical trial conducted in the Department of Periodontology at a tertiary care dental institution. The study protocol was reviewed and approved by the Institutional Ethics Committee and all procedures were conducted in accordance with ethical standards.

## Sample size and study population:

A total of 100 patients diagnosed with chronic periodontitis were included in the study. Patients were selected from those reporting to the outpatient department.

## Inclusion criteria:

[1] Patients aged between 25 and 60 years

[2] Presence of chronic periodontitis with probing pocket depth ≥5 mm

[3] Systemically healthy individuals

[4] Patients willing to participate and provide informed consent

## Exclusion criteria:

[1] Patients with systemic diseases affecting periodontal healing (e.g., diabetes mellitus)

[2] Smokers and tobacco users

[3] Pregnant or lactating women

[4] Patients who had undergone periodontal therapy in the past 6 months

[5] Use of antibiotics or anti-inflammatory drugs within the last 3 months 

## Pre-Surgical phase:

All patients underwent initial periodontal therapy, including:

[1] Scaling and root planing

[2] Oral hygiene instructions

[3] Revaluation after 4 weeks

Baseline clinical parameters were recorded at this stage.

## Clinical parameters assessed:

The following parameters were recorded at baseline, 1 month and 3 months:

[1] Plaque Index (PI)

[2] Gingival Index (GI)

[3] Probing Pocket Depth (PPD)

[4] Clinical Attachment Level (CAL)

[5] Post-operative pain (using Visual Analog Scale)

[6] Wound healing index

## Surgical procedure:

## Group A: Microsurgical approach: 

[1] Procedures were performed under magnification using surgical loupes/microscope

[2] Microsurgical instruments were used

[3] Minimal flap reflection with precise incisions

[4] Atraumatic tissue handling

[5] Suturing performed using fine sutures (6-0 or 7-0)

## Group B: Conventional approach:

[1] Standard flap surgery performed without magnification

[2] Conventional instruments used

[3] Routine flap reflection and debridement

[4] Suturing performed using standard sutures (3-0 or 4-0)

## Post-operative care:

[1] Analgesics prescribed for pain management

[2] Chlorhexidine mouthwash (0.12%) twice daily for 2 weeks

[3] Sutures removed after 7-10 days

[4] Patients were instructed to maintain oral hygiene

## Statistical analysis:

Data were analyzed using statistical software. Mean and standard deviation were calculated for all variables. Intragroup comparisons were performed using paired t-test. Intergroup comparisons were performed using independent t-test. A p-value < 0.05 was considered statistically significant.

## Results:

A total of 100 patients completed the study, with 50 patients in each group: Group A: Microsurgical approach and Group B: Conventional approach. All patients were evaluated at baseline, 1 month and 3 months post-operatively. No dropouts were recorded. Both groups showed statistically significant improvements from baseline (p < 0.05). However, microsurgical group demonstrated greater reduction in PPD (50.8% vs 40.6%), Greater clinical attachment gain (44.4% vs 32.4%) and the Differences were statistically significant (p < 0.05). These findings are consistent with studies showing enhanced precision and tissue preservation with microsurgical techniques (Table 1). Microsurgical group showed 53% lower pain scores. Analgesic consumption reduced by ~57%. Excellent healing (score 4) observed in 92% vs 48%. These results strongly support that microsurgery enhances early wound healing and reduces post-operative discomfort, as also demonstrated in recent randomized trial (Table 2). Gingival recession increase was significantly lower in Group A (microsurgical group) compared to Group B, with a mean increase of 0.3 ± 0.2 mm versus 0.7 ± 0.3 mm (p = 0.01). Patient satisfaction scores were significantly higher in Group A (8.9 ± 0.7) compared to Group B (6.5 ± 1.0; p < 0.001). Esthetic outcomes were also significantly superior in Group A, with 88% reporting excellent results versus 52% in Group B (p < 0.001). The reduced recession and improved esthetics are attributed to atraumatic tissue handling and precise suturing, key principles of microsurgery (Table 3).

## Discussion:

The current randomized controlled trial compared the clinical effectiveness of the microsurgical flap surgery with conventional periodontal flap surgery in 100 patients. These results indicate that although both methods are effective in enhancing periodontal parameters, the microsurgical technique has better outcomes in various aspects. Among the most interesting results of this research was the much stronger decrease in probing pocket depth (PPD) and increase in clinical attachment level (CAL) in the microsurgical group. This can be blamed on better visualization and accuracy with the use of magnification. Microsurgical methods enable superior recognition and elimination of the granulation tissue and calculus deposits and this enhances the treatment outcomes. They found similar results in a limited number of studies in which they established better clinical parameters in cases of microsurgical flap operations [10, 11]. Nonetheless, there are studies that have shown no statistically significant difference between the use of microsurgical and conventional methods on the basis of PPD and CAL reduction. Siaili *et al.* (2017) discovered that both methods yielded better clinical outcomes, but at 3 months, there were no significant differences between the groups [12]. The difference can be attributed to variations in sample size, period of follow-up and operator competence. One of the benefits of the microsurgical technique that was noted during this research was the high early wound healing. In the current study, 92 percent of microsurgical sites had excellent healing as opposed to 48 percent in the conventional group. These results are in accordance with the past studies which have indicated that microsurgery is beneficial in terms of primary wound healing and reduction of tissue damage. The high rate of healing is mainly attributed to accurate location of incisions, fewer flaps that are reflected and use of fine sutures which minimize tension on the tissues [13, 14]. Another essential parameter that affects patient acceptance of surgical procedures is the post-operative pain. The microsurgical group had much less pain scores and less analgesic use in this study. These results are corroborated by previous experiments which had also indicated that there is minimal post-operative discomfort with the use of microsurgical techniques because of minimal tissue manipulations and inflammations [15]. A frequent side effect of periodontal flap surgery is gingivitis recession. The current research showed a great reduction in gingivial recession in the microsurgical group. This can be explained by a better adaptation of the flaps and the maintenance of blood supply. Less trauma during flap elevation assists in preserving vascular integrity, necessary to achieve optimal healing. Microsurgical group also had a high patient satisfaction and esthetic outcome. The contemporary periodontal therapy is more focused on how it can be used to achieve functional results but also patient-focused parameters, e.g., comfort and esthetics. Microsurgery meets these needs through its ability to offer less invasive treatment with better cosmetic outcomes. The recent developments of periodontal microsurgery have added more strength to its clinical usage. Research findings on gingivolinggival grafting and regenerative surgery have demonstrated enhanced color match, less edema and enhanced tissue integration with the use of microsurgical procedures [16]. The only weakness found with such studies however is the longer operative time and special equipment and training required. Although it has many benefits, the microsurgical method has some weaknesses. It needs more training, more expensive equipment and steep learning curve. Furthermore, the advantages of microsurgery could be operator-specific and this can affect reproducibility of outcomes. The other factor that is critical to keep in mind is that this study did not evaluate long-term outcomes that were more than 6 months. Although the healing and clinical outcomes are encouraging in the short-term, additional research using long-term follow-ups is needed to determine the long-term results. In general, the results of this research indicate that microsurgical periodontal flap surgery is a better option as compared to the traditional methods, especially regarding the comfort of the patient, healing and esthetic results. But both methods are clinically viable and the selection of the method might be based on clinical expertise, equipment availability and patient preference.

## Conclusion:

Microsurgical and conventional periodontal flap procedures were both useful in enhancing the clinical periodontal parameters. The microsurgical method, however, showed better results in the area of probing pocket depth reduction, clinical attachment gain, wound healing, post-operative pain reduction and patient satisfaction. Consequently, it can be assumed that the use of the microsurgical methods as the alternative to the traditional periodontal flap surgery is more sophisticated and less traumatizing to the patient.

## Figures and Tables

**Table 1:** Comparison of clinical parameters (Baseline vs 3 Months)

**Parameter**	**Group A (Microsurgery) Baseline**	**Group A 3 Months**	**% Change**	**Group B (Conventional) Baseline**	**Group B 3 Months**	**% Change**	**p-value**
Plaque Index (PI)	2.10 ±0.30	0.90 ±0.20	↓57.1%	2.08 ±0.28	1.05 ±0.25	↓49.5%	0.03
Gingival Index (GI)	2.25 ±0.35	0.95 ±0.22	↓57.8%	2.20 ±0.30	1.10 ±0.30	↓50.0%	0.04
Probing Pocket Depth (PPD, mm)	6.5 ±0.8	3.2 ±0.6	↓50.8%	6.4 ±0.7	3.8 ±0.7	↓40.6%	0.01
Clinical Attachment Level (CAL, mm)	7.2 ±0.9	4.0 ±0.7	↑44.4% gain	7.1 ±0.8	4.8 ±0.8	↑32.4% gain	0.02

**Table 2:** Post-operative pain and healing outcomes

**Parameter**	**Group A (Microsurgery)**	**Group B (Conventional)**	**p-value**
Mean VAS Pain Score (Day 3)	2.1 ±0.8	4.5 ±1.2	<0.001
Analgesic Consumption (No. of tablets)	2.2 ±1.0	5.1 ±1.5	<0.001
Early Healing Index (Day 7) Score 4 (%)	92%	48%	<0.001

**Table 3:** Gingival recession and patient satisfaction

**Parameter**	**Group A**	**Group B**	**p-value**
Gingival Recession Increase (mm)	0.3 ±0.2	0.7 ±0.3	0.01
Patient Satisfaction Score (1-10)	8.9 ±0.7	6.5 ±1.0	<0.001
Esthetic Outcome (Excellent %)	88%	52%	<0.001
